# The Effects of Ru^4+^ Doping on LiNi_0.5_Mn_1.5_O_4_ with Two Crystal Structures

**DOI:** 10.3390/ma15124273

**Published:** 2022-06-16

**Authors:** Xinli Li, Ben Su, Wendong Xue, Junnan Zhang

**Affiliations:** 1 School of Materials Science and Engineering, University of Science and Technology Beijing, Beijing 100083, China; xinbattery@163.com (X.L.); suben111@163.com (B.S.); 2 Shandong Wina Green Power Technology Co., Ltd., Weifang 261000, China; jnzhang@winabattery.com

**Keywords:** solid-state reactions, LiNi_0.5_Mn_1.5_O_4_, Ru^4+^, space group, comparative study

## Abstract

Doping of Ru has been used to enhance the performance of LiNi_0.5_Mn_1.5_O_4_ cathode materials. However, the effects of Ru doping on the two types of LiNi_0.5_Mn_1.5_O_4_ are rarely studied. In this study, Ru^4+^ with a stoichiometric ratio of 0.05 is introduced into LiNi_0.5_Mn_1.5_O_4_ with different space groups (Fd$\bar{3}$m, P4_3_32). The influence of Ru doping on the properties of LiNi_0.5_Mn_1.5_O_4_ (Fd$\bar{3}$m, P4_3_32) is comprehensively studied using multiple techniques such as XRD, Raman, and SEM methods. Electrochemical tests show that Ru^4+^-doped LiNi_0.5_Mn_1.5_O_4_ (P4_3_32) delivers the optimal electrochemical performance. Its initial specific capacity reaches 132.8 mAh g^−1^, and 97.7% of this is retained after 300 cycles at a 1 C rate at room temperature. Even at a rate of 10 C, the capacity of Ru^4+^-LiNi_0.5_Mn_1.5_O_4_ (P4_3_32) is still 100.7 mAh g^−1^. Raman spectroscopy shows that the Ni/Mn arrangement of Ru^4+^-LiNi_0.5_Mn_1.5_O_4_ (Fd$\bar{3}$m) is not significantly affected by Ru^4+^ doping. However, LiNi_0.5_Mn_1.5_O_4_ (P4_3_32) is transformed to semi-ordered LiNi_0.5_Mn_1.5_O_4_ after the incorporation of Ru^4+^. Ru^4+^ doping hinders the ordering process of Ni/Mn during the heat treatment process, to an extent.

## 1. Introduction

The continuous development of lithium-ion batteries has driven the progress of the electric vehicle industry. However, the cruising mileage of electric vehicles still has a great deal of room for improvement. Energy density has always been one of the key factors limiting the range. From the formula *W = Q • U*, we know that increasing the specific capacity and discharge voltage of the cathode material is the main way to increase the power density of lithium-ion batteries (LIBs). Spinel LiNi_0.5_Mn_1.5_O_4_ (LNMO) is considered to be one of the most promising candidates for high-power lithium-ion battery systems, and this is attributed to its ultra-fast 3D Li^+^ diffusion speed, good high-rate capability, excellent cyclic stability, low cost, and environmental friendliness. LiNi_0.5_Mn_1.5_O_4_ has a high discharge platform (vs. Li/Li^+^ ≈ 4.7 V) [1,2,3] and a theoretical discharge capacity of 146.7 mAh g^−1^; therefore, it has a fairly high theoretical energy density [4].

Built on the arrangement of Mn^4+^ and Ni^2+^ in the crystal lattice, LiNi_0.5_Mn_1.5_O_4_ can have two crystal structures: a face-centered cubic structure (Fd$\bar{3}$m) or a simple cubic structure (P4_3_32). The former is a disordered structure in which Mn^4+^ and Ni^2+^ ions are randomly distributed on the octahedral 16d sites. The latter has an ordered structure in which Mn^4+^ ions occupy the 12d positions and Ni^2+^ ions occupy the 4a sites [5,6]. In addition, almost no Mn^3+^ ions are generated in the crystal structure. It is generally believed that disordered LiNi_0.5_Mn_1.5_O_4_ containing Mn^3+^ has higher electronic conductivity and lithium-ion conductivity, and thus has better high-rate performance [7,8,9,10]. However, the presence of too many Mn^3+^ ions in LiNi_0.5_Mn_1.5_O_4_ will damage the cyclic stability of the cathode material, because Mn^3+^ is prone to the disproportionation reaction 2Mn^3+^ = Mn^2+^ + Mn^4+^. Generated Mn^2+^ ions dissolve in the electrolyte, migrate to the anode electrode, and further deposit on the surface of the anode electrode. The continuous disproportionation–dissolution–migration–deposition behavior causes irreversible loss of battery capacity [11,12,13]. However, the ordered LiNi_0.5_Mn_1.5_O_4_ also has the problem of poor cycling stability. Owing to the two-step phase transition occurring during the charge/discharge process, the structural stability of LiNi_0.5_Mn_1.5_O_4_ (P4_3_32) is highly susceptible and prone to irreversible structural damage. Thus, LiNi_0.5_Mn_1.5_O_4_ with a small amount Mn^3+^ and a structure that is not completely ordered may have optimal properties in actual usage. When the calcination temperature is increased above 700 °C, a disordered phase is formed accompanied by the loss of oxygen and the appearance of Mn^3+^ inside the crystal lattice. The arrangement state of the transition metal ions can be controlled by an annealing process [14]. The oxygen defects and Mn^3+^ can be eliminated by annealing at 700 °C, and a disordered state can be transformed into an ordered state [15,16,17,18].

Due to poor uniformity of the raw material mixture, LiNi_0.5_Mn_1.5_O_4_ synthesized by the conventional solid-state method may contain rock-salt impurities and deliver a low specific capacity. To enhance the electrochemical performance of LiNi_0.5_Mn_1.5_O_4_, one efficient strategy is to dope with transition metal ions such as Na [19], Mg [20], Er [21], Fe [22], Ga [23], Ru [24], Cr [25], and Al [26] in the framework, to improve conductivity. Jianbing G. et al. studied the effects of Ru doping and the doping amount on the properties of LiNi_0.5_Mn_1.5_O_4_ cathode material [27,28]. The electrochemical performance of LiNi_0.5_Mn_1.5_O_4_ improved greatly after Ru doping; the initial discharge specific capacity increased from 103 mAh g^−1^ to 125.3 mAh g^−1^ and the 5 C-rate capacity increased by about 30 mAh g^−1^. However, previous studies have usually been carried out on LiNi_0.5_Mn_1.5_O_4_ (Fd$\bar{3}$m) [24,29], and few studies have compared the effects of transition metal doping on the two types of cathode materials. In this study, LiNi_0.5_Mn_1.5_O_4_ cathode materials with Fd$\bar{3}$m and P4_3_32 space groups were synthesized through a combination of typical solid-state reactions and heat treatment processes. Multiple techniques such as XRD, Raman and SEM methods were utilized to comprehensively study the influence of Ru^4+^ doping modification on the microstructure, micromorphology, and electrochemical performance of LiNi_0.5_Mn_1.5_O_4_ with the two space groups.

## 2. Materials and Methods

### 2.1. Material Synthesis

LiNi_0.5_Mn_1.5_O_4_ with different structures was synthesized via traditional solid-state reactions. A typical route was as follows: (1) Li_2_CO_3_, NiCO_3_, and MnCO_3_ in a stoichiometric ratio of 0.525:0.5:1.5 were mixed in alcohol; (2) a process of ball milling was performed at a speed of 400 r/min for 4 h, and the obtained mixture was completely dried at 60 °C and subsequently pulverized; (3) the powder was heated to 900 °C in a tube furnace at a rate of 5 °C/min and held for 12 h, followed by cooling naturally to room temperature to obtain the LiNi_0.5_Mn_1.5_O_4_ with space group of Fd$\bar{3}$m, denoted LNMO (Fd$\bar{3}$m). Alternatively, the heated material was insulated at 700 °C for 48 h and cooled naturally to room temperature to give the P4_3_32-structured LiNi_0.5_Mn_1.5_O_4_, denoted LNMO (P4_3_32). Li_2_CO_3_, NiCO_3_, MnCO_3_, and RuO_2_ were mixed in a stoichiometric ratio of 0.525:0.45:1.5:0.05, and the above steps were repeated to obtain Ru-doped LiNi_0.5_Mn_1.5_O_4_ with different structures, denoted Ru^4+^-LNMO (Fd$\bar{3}$m) and Ru^4+^-LNMO (P4_3_32), respectively.

### 2.2. Characterization

To investigate the influence of the Ru^4+^ doping on the crystal structure of LiNi_0.5_Mn_1.5_O_4_ with different space groups, X-ray diffraction (XRD, Ultima IV, Tokyo, Japan) was carried out using Cu Kα radiation in the range 10° ≤ *2θ* ≤ 70°. The morphologies of Ru-doped LiNi_0.5_Mn_1.5_O_4_ with different structures were recorded using scanning electron microscopy (SEM, FESEM Quanta TEG 450, Hillsboro, OR, USA). The phase structures of LiNi_0.5_Mn_1.5_O_4_ with and without doping for the different structures were investigated using a Renishaw inVia plus-type micro-Raman spectrometer.

### 2.3. Preparation of Electrodes and Construction of Cells

LiNi_0.5_Mn_1.5_O_4_ (80 wt%) was mixed with 10 wt% acetylene black and 10 wt% polyvinylidene fluoride (PVDF) in the appropriate amount of N-Methyl pyrrolidone (NMP). The obtained slurry was coated onto aluminum foil, then dried at 80 °C for 2 h in air and at 120 °C for 8 h in a vacuum oven. Finally, a Celgard 2400 argon-filled glove box was used as the separator to assemble coin-type LiNi_0.5_Mn_1.5_O_4_/Li cells.

### 2.4. Electrochemical Measurements

The electrochemical properties of LiNi_0.5_Mn_1.5_O_4_ before and after Ru^4+^ doping were measured with CR2025-type coin cells (HF-Kejing, Hefei, China). All the charge-discharge behaviors were evaluated at a rate of 1 C at room temperature, utilizing a LAND battery testing system. The rate of 1 C was set at 147 mA g^−1^, and the current densities for testing were determined on the basis of the weight of cathode material. In the evaluation of cycle performance, the cells were charged and discharged in the voltage range of 3.5 V to 5.0 V for 300 cycles. The rate tests were conducted at rates of 0.2 C, 0.5 C, 1 C, 2 C, 5 C, and 10 C and then reversed successively back to 0.2 C.

## 3. Results and Discussion

**Figure 1:**
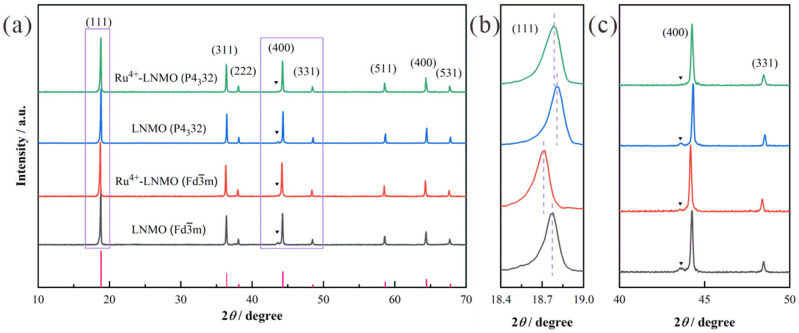
(**a**) XRD patterns of LiNi_0.5_Mn_1.5_O_4_ (Fd$\bar{3}$m, P4_3_32) before and after Ru^4+^ doping; (**b**) the enlarged (111) diffraction peak in the XRD patterns; (**c**) the enlarged impurities diffraction peak in the XRD patterns.

The X-ray diffraction patterns of the LiNi_0.5_Mn_1.5_O_4_ (Fd$\bar{3}$m, P4_3_32), with and without Ru^4+^ doping, are compared in Figure 1. Clearly, all peaks of the four as-prepared samples are in agreement with the XRD patterns of typical spinel LiNi_0.5_Mn_1.5_O_4_. Ru^4+^ doping does not change the primary lattice framework of the LiNi_0.5_Mn_1.5_O_4_. Since the radius of Ru^4+^ is comparable to that of Ni^2+^ [30], a slight distortion of the lattice will be caused by the introduction of Ru^4+^. This is also reflected in the diffraction patterns, with the (111) peaks of Ru^4+^-LiNi_0.5_Mn_1.5_O_4_ (Fd$\bar{3}$m, P4_3_32) all shifting slightly towards the low-angle region. Furthermore, the larger lattice parameters *a* and *c* facilitate the diffusion of Li^+^ and subsequently enhance the high-rate capability of the battery. The weak peaks (2*θ* at ≈37.5°, 43.6°, 47.5°, and 63.5°) correspond to the rock-salt impurity phase Li_x_Ni_1−x_O, which is mainly caused by oxygen loss [31,32]. In terms of traditional solid-state methods, insufficient mixing and high sintering temperatures inevitably contribute to the volatilization of Ni/Li and the formation of Li_x_Ni_1-x_O components. Amplifying a partial area (2*θ* = 40°~50°), it can be found that the weak peaks of the impurity basically disappear in the spectrum of Ru^4+^-LiNi_0.5_Mn_1.5_O_4_ (P4_3_32), but tiny impurity peaks still emerge in the spectrum of Ru^4+^-LiNi_0.5_Mn_1.5_O_4_ (Fd$\bar{3}$m). This demonstrates that Ru^4+^ doping has a noticeable effect in eliminating Li_x_Ni_1−x_O-like impurity phases for LiNi_0.5_Mn_1.5_O_4_ (both Fd$\bar{3}$m and P4_3_32) and stabilizing the spinel crystal structure.

**Figure 2:**
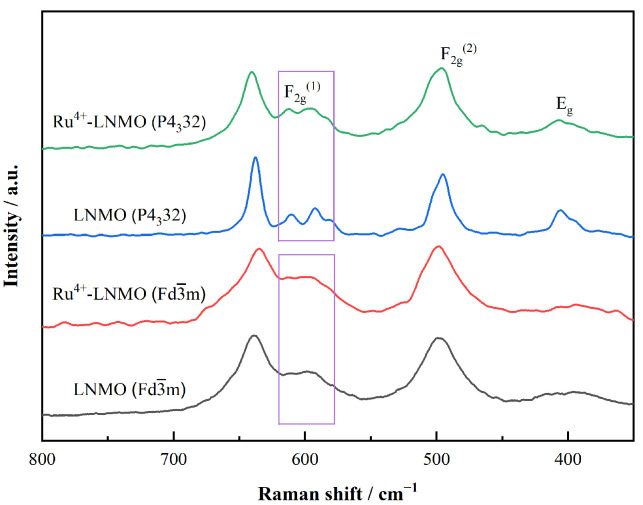
Raman spectra of the LiNi_0.5_Mn_1.5_O_4_ (Fd$\bar{3}$m, P4_3_32) before and after Ru^4+^ doping.

The Raman spectra of LiNi_0.5_Mn_1.5_O_4_ with different structures before and after doping are shown in Figure 2. The peaks at around 630 cm^−1^ are assigned to the symmetric Mn-O stretching vibration, and the peaks at around 482 cm^−1^ correspond to the Ni-O stretching mode. The splitting of the F_2g_^(1)^ vibration mode near 580–600 cm^−1^ is clear evidence for the ordered structure, while a lack of F_2g_^(1)^ splitting corresponds to the disordered state. It is generally accepted that a higher degree of disorder indicates a higher Mn^3+^ content. It can be observed that Ru^4+^-LiNi_0.5_Mn_1.5_O_4_ (Fd$\bar{3}$m) and LiNi_0.5_Mn_1.5_O_4_ (Fd$\bar{3}$m) have highly similar F_2g_^(1)^ vibration modes, in which no obvious splitting peaks appear. Ru^4+^ doping does not affect the arrangement of Ni/Mn in the lattice. Ru^4+^-LiNi_0.5_Mn_1.5_O_4_ (Fd$\bar{3}$m) still maintains a highly Ni/Mn disordered state. Nevertheless, the splitting degree of Ru^4+^-LiNi_0.5_Mn_1.5_O_4_ (P4_3_32) is much less than the obvious splitting in the LiNi_0.5_Mn_1.5_O_4_ (P4_3_32) sample, indicating that Ru doping enhances the degree of disorder of LiNi_0.5_Mn_1.5_O_4_ (P4_3_32) and enhances the content of Mn^3+^ ions to some extent. During the process of heat treatment, the introduction of Ru^4+^ inhibits the Ni/Mn ordering process so that ordered LiNi_0.5_Mn_1.5_O_4_ is transformed to semi-ordered LiNi_0.5_Mn_1.5_O_4_. Previous studies have demonstrated that the Ni/Mn arrangement is mainly related to the radius and valence of nickel/manganese ions. LiNi_0.5_Mn_1.5_O_4_ may have disordered (space group: Fd$\bar{3}$m) or ordered (space group: P4_3_32) spinel structures. In the ordered structure, Ni occupies the 4b octahedral sites and Mn occupies the 12d octahedral sites. Ni and Mn are randomly distributed among the 16d octahedral sites in the disordered LiNi_0.5_Mn_1.5_O_4_ structure. In this study, Ru^4+^ was utilized to replace the corresponding content of N^i2+^ in the lattice structures. However, Ru^4+^ and Mn^4+^ have same valence state and similar ionic radii [30], and this can easily cause cationic mixing of Ru/Mn. Ru^4+^ can tend to occupy the 12c sites of Mn^4+^, causing the substituted Mn^4+^ to compete with Ni^2+^ for the 4b sites. By substituting Ru^4+^ with a high valence state for Ni^2+^ with a low valence state, some of the Mn^4+^ ions are reduced to Mn^3+^ for the sake of charge compensation, thereby increasing the cationic mixing degree.

It is generally believed that the crystal plane in contact with the electrolyte and the particle size both have an important influence on the electrochemical performance of LiNi_0.5_Mn_1.5_O_4_ cathode material [33,34,35,36]. The dissolution of the transition metal is closely linked to the stability of the interface. For example, Mn^2+^ derived from the disproportionation reaction is most easily dissolved from the {110} crystal plane into the electrolyte. Therefore, inhibiting the growth of the {110} crystal plane can effectively reduce the dissolution of the transition metal and thus improve the cyclic stability [37]. Compared with the {110} planes, the {100} crystal planes have a positive effect on the electrochemical performance [38,39]. Particle size is another important factor affecting stability. Nanoscale LiNi_0.5_Mn_1.5_O_4_ has shorter Li^+^ diffusion paths, but at the same time, the larger specific surface area also leads to more serious side reactions at the interface and instability of the battery system [40,41]. Large (micron level) LiNi_0.5_Mn_1.5_O_4_ particles have a smaller specific surface area, which effectively reduces the degree of side reactions to enhance cycling performance.

**Figure 3:**
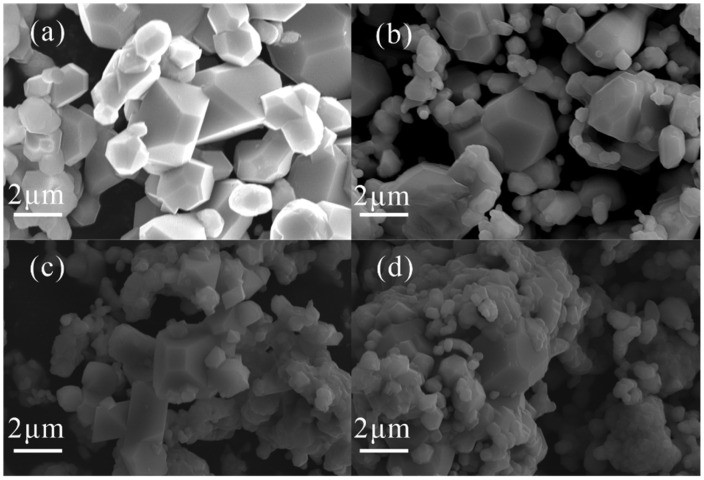
SEM images of the LiNi_0.5_Mn_1.5_O_4_ (Fd$\bar{3}$m, P4_3_32) before and after Ru^4+^doping. (**a**) LiNi_0.5_Mn_1.5_O_4_ (Fd$\bar{3}$m); (**b**) LiNi_0.5_Mn_1.5_O_4_ (P4_3_32); (**c**) Ru^4+^-LiNi_0.5_Mn_1.5_O_4_ (Fd$\bar{3}$m); (**d**) Ru^4+^-LiNi_0.5_Mn_1.5_O_4_ (P4_3_32).

The micro-morphology of the LiNi_0.5_Mn_1.5_O_4_ (Fd$\bar{3}$m, P4_3_32) before and after Ru^4+^ doping is shown in Figure 3. Ru^4+^-LiNi_0.5_Mn_1.5_O_4_ (Fd$\bar{3}$m) particles have a truncated octahedral morphology, and Ru^4+^-LiNi_0.5_Mn_1.5_O_4_ (P4_3_32) particles have a spherical truncated polyhedron morphology. Additionally, both of them have a certain particle size distribution. Some particle diameters are about 2 μm, and a large number of particle diameters are about 1 μm. After doping, grains of LiNi_0.5_Mn_1.5_O_4_ with different structures have no obvious distortion or morphological changes. However, strictly speaking, the particle growth of the active material seems to be repressed by Ru^4+^ cooperation during the calcination process, which reduces the final particle size to some extent.

**Figure 4:**
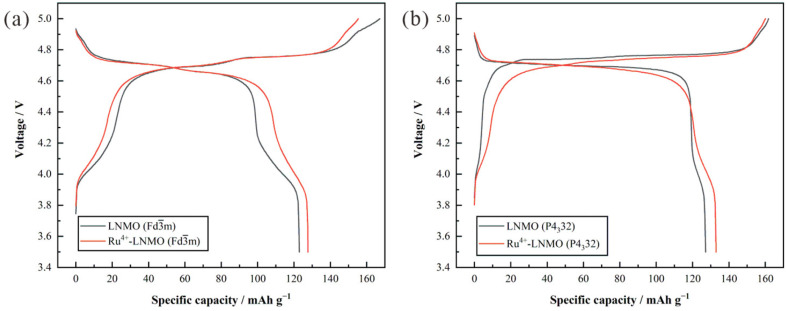
Initial charge–discharge curves of (**a**) LiNi_0.5_Mn_1.5_O_4_ (Fd$\bar{3}$m) and (**b**) LiNi_0.5_Mn_1.5_O_4_ (P4_3_32) before and after Ru^4+^ doping in the voltage range of 3.5 V~5.0 V at a rate of 0.2 C.

The initial charge–discharge curves of the LiNi_0.5_Mn_1.5_O_4_ (Fd$\bar{3}$m, P4_3_32) before and after doping in the voltage range of 3.5~5.0 V at a rate of 0.2 C are shown in Figure 4. Compared with the discharge capacity (123.0 mAh g^−1^) of LiNi_0.5_Mn_1.5_O_4_ (Fd$\bar{3}$m), the capacity of Ru^4+^-LiNi_0.5_Mn_1.5_O_4_ (Fd$\bar{3}$m) is increased to 127.7 mAh g^−1^. Meanwhile, the 4.1 V platform capacity and the capacity ratio are reduced from 18.9 mAh g^−1^/15.4% to 14.1 mAh g^−1^/11.0%, respectively. Compared to LiNi_0.5_Mn_1.5_O_4_ (P4_3_32), the 4.1V platform capacity (9.3 mAh g^−1^, 7.07%) of Ru^4+^-LiNi_0.5_Mn_1.5_O_4_ (P4_3_32) has more than doubled, and the discharge capacity has reached the maximum value. In addition, the average valence of the transition metal nickel ions may decrease with the introduction of Ru^4+^, resulting in an increase in the 4.7 V platform discharge capacity. On the other hand, Ru^4+^ doping can also prevent the sample from reacting with oxygen during the heat treatment process, which is manifested by the presence of a certain amount of Mn^3+^. The presence of Mn^3+^ can further increase the discharge capacity of the material. Therefore, the discharge capacity of Ru^4+^-LiNi_0.5_Mn_1.5_O_4_ (P4_3_32) reaches the maximum value among the four samples.

**Figure 5:**
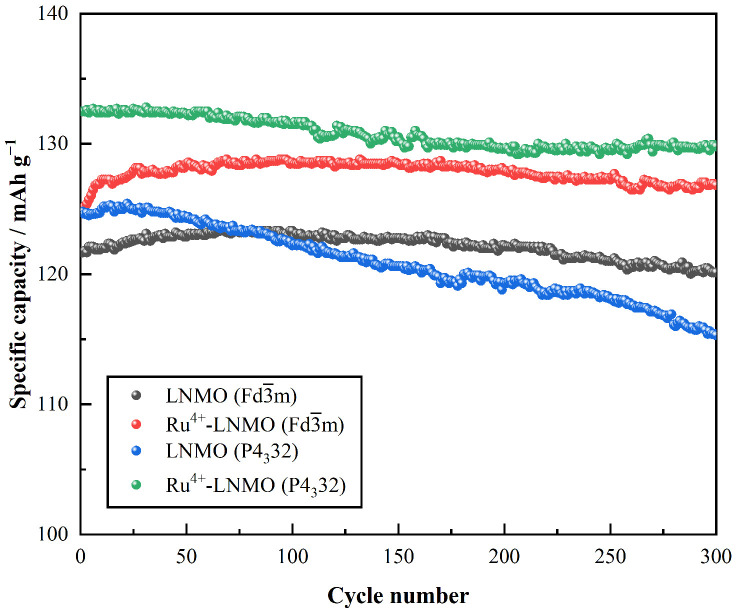
Cycling performance of the LiNi_0.5_Mn_1.5_O_4_ (Fd$\bar{3}$m, P4_3_32) before and after Ru^4+^doping at a 1 C rate in the voltage range of 3.5 V~5.0 V at 25 °C.

The LiNi_0.5_Mn_1.5_O_4_/Li half cells were all pre-cycled at 0.2 C for three cycles. Figure 5 shows the cycling performance of cells at the 1 C rate (147.0 mA g^−1^) in the voltage range of 3.5 V to 5.0 V (vs. Li/Li^+^) at room temperature. It can be observed that the Ru^4+^-LiNi_0.5_Mn_1.5_O_4_ (Fd$\bar{3}$m) delivers a higher discharge capacity than LiNi_0.5_Mn_1.5_O_4_ (Fd$\bar{3}$m), with an initial specific capacity increase from 121.7 mAh g^−1^ to 124.8 mAh g^−1^. In addition, the capacity retention after 300 cycles is also increased from 97.3% to 98.5%. On the other hand, the LiNi_0.5_Mn_1.5_O_4_ (P4_3_32) sample shows an initial discharge capacity of 125.4 mAh g^−1^ but delivers a lower retention of 92.0%. With Ru^4+^ doping, the initial capacity of Ru^4+^-LiNi_0.5_Mn_1.5_O_4_ (P4_3_32) reaches the highest value of 132.8 mAh g^−1^, and the capacity retention after 300 cycles also recovers to 97.8%.

**Figure 6:**
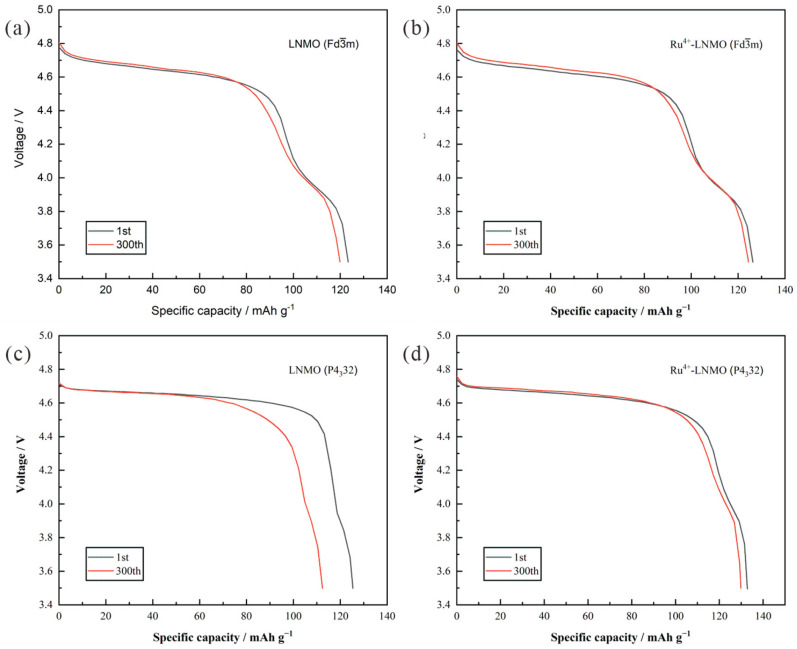
Discharge profiles of the LiNi_0.5_Mn_1.5_O_4_ (Fd$\bar{3}$m, P4_3_32) before and after Ru^4+^ doping from 3.5 V to 5.0 V at a 1 C rate in the 1st and 300th cycle. (**a**) LiNi_0.5_Mn_1.5_O_4_ (Fd$\bar{3}$m); (**b**) LiNi_0.5_Mn_1.5_O_4_ (P4_3_32); (**c**) Ru^4+^-LiNi_0.5_Mn_1.5_O_4_ (Fd$\bar{3}$m); (**d**) Ru^4+^-LiNi_0.5_Mn_1.5_O_4_ (P4_3_32).

The discharge voltage curves in the 1st and 300th cycles of the four samples at the 1C rate (147.0 mAh g^−1^) at room temperature are displayed in Figure 6. Before and after 300 cycles, the curves for LiNi_0.5_Mn_1.5_O_4_ (Fd$\bar{3}$m) have a high degree of coincidence, but careful observation shows that both the 4.7 V and 4.1 V platforms are shortened slightly. Ni^2+^ and Mn^3+^ dissolve into the electrolyte, causing a loss of battery capacity. The introduction of Ru^4+^ inhibits the dissolution of Ni^2+^ and Mn^3+^ during the charge–discharge process by stabilizing the crystal structure. Thus, the plateau-shortening degree of Ru^4+^-LiNi_0.5_Mn_1.5_O_4_ (Fd$\bar{3}$m) is slightly less than that of LiNi_0.5_Mn_1.5_O_4_ (Fd$\bar{3}$m). In addition, LiNi_0.5_Mn_1.5_O_4_ (P4_3_32) as a cathode material suffers severe capacity attenuation. The discharge voltage curves in the 1st and 300th cycles are significantly dissimilar, reflecting the observable shortening of the 4.7 V platform. Compared to LiNi_0.5_Mn_1.5_O_4_ (P4_3_32), Ru^4+^-LiNi_0.5_Mn_1.5_O_4_ (P4_3_32) delivers excellent cyclic stability after 300 cycles. Ru^4+^ plays a major role in improving the cyclic stability. The Ru-O bond energy is higher than those of the Ni-O bond and the Mn-O bond, which can stabilize the crystal structure to reduce the lattice damage. Based on the analysis of the Raman spectroscopy results, Ru^4+^-LiNi_0.5_Mn_1.5_O_4_ (P4_3_32) transforms to semi-ordered LiNi_0.5_Mn_1.5_O_4_ and contains a certain amount of Mn^3+^. The octahedral distortion caused by Mn^3+^ promotes the generation of more Li^+^ diffusion channels in active materials, which is beneficial to the cycling stability of the electrode. Therefore, Ru^4+^-LiNi_0.5_Mn_1.5_O_4_ (P4_3_32) not only has superior capacity but also has considerable cyclic stability.

**Figure 7:**
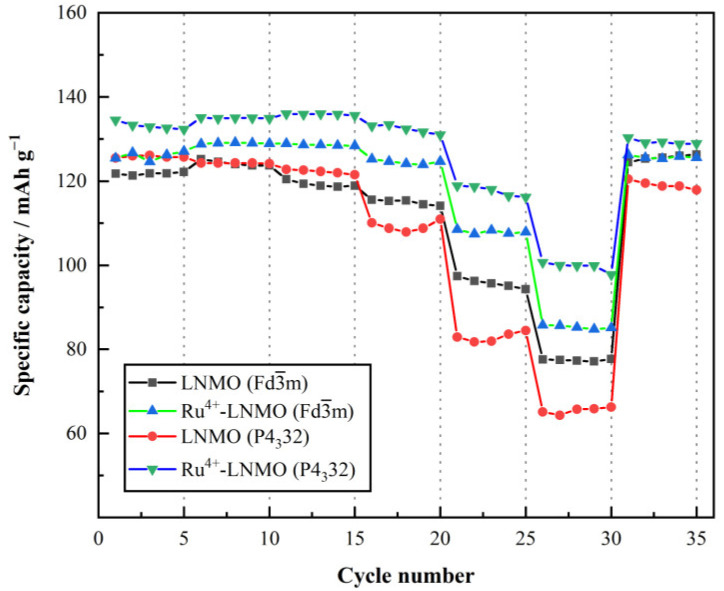
Rate performance of LiNi_0.5_Mn_1.5_O_4_ (Fd$\bar{3}$m, P4_3_32) before and after Ru^4+^ doping.

The rate tests for LiNi_0.5_Mn_1.5_O_4_ (Fd$\bar{3}$m, P4_3_32) before and after Ru^4+^doping at different current densities were carried out in the voltage range of 3.5 V to 5.0 V, as shown in Figure 7. At rates of 0.2 C, 0.5 C, 1 C, 2 C, 5 C, and 10 C, Ru^4+^-LiNi_0.5_Mn_1.5_O_4_ (Fd$\bar{3}$m) and Ru^4+^-LiNi_0.5_Mn_1.5_O_4_ (P4_3_32) both demonstrate higher discharge capacities than LiNi_0.5_Mn_1.5_O_4_ (Fd$\bar{3}$m, P4_3_32), and Ru^4+^-LiNi_0.5_Mn_1.5_O_4_ (P4_3_32) delivers the optimum rate capacity. The effect of Ru^4+^ doping on the high-rate performance for ordered LiNi_0.5_Mn_1.5_O_4_ is more noticeable at high rates. Upon increasing the current intensity, the superiority becomes particularly evident. All samples deliver a decrease in specific discharge capacity when the imposed current density increases from a rate of 0.2 C (29.4 mA g^−1^) to a rate of 5 C (735 mA g^−1^). Ru^4+^-LiNi_0.5_Mn_1.5_O_4_ (P4_3_32) shows a discharge capacity retention of ≈89.4% (≈118 mAh g^−1^ at 5 C; ≈132 mAh g^−1^ at 0.2 C), compared with ≈85.3% retention (≈108.3 mAh g^−1^ at 5 C; ≈126.2 mAh g^−1^ at 0.2 C) for Ru^4+^-LiNi_0.5_Mn_1.5_O_4_ (Fd$\bar{3}$m). Upon increasing the current intensity to a rate of 10 C (1470 mA g^−1^), the capacity retention of Ru^4+^-LiNi_0.5_Mn_1.5_O_4_ (P4_3_32) still remains at ≈75.8%, compared with ≈67.2% for Ru^4+^-LiNi_0.5_Mn_1.5_O_4_ (Fd$\bar{3}$m). Meanwhile, the capacities decay to only ≈63.4% and 52.8% of the initial capacities for LiNi_0.5_Mn_1.5_O_4_ (Fd$\bar{3}$m) and LiNi_0.5_Mn_1.5_O_4_ (P4_3_32), respectively.

The superior rate performance of Ru^4+^-LiNi_0.5_Mn_1.5_O_4_ (P4_3_32) is mainly related to rapid migration rate of Li^+^ during the charge/discharge process. Firstly, owing to the incorporation of Ru^4+^, the lattice parameters of LiNi_0.5_Mn_1.5_O_4_ increase, which may widen the Li^+^ movement path and reduce the activation energy required for the migration of Li^+^. Secondly, the electron popping path changes from an O-Ni-O-Ni route to an O-Ru/Ni-O-Ru/Ni route, which facilitates the transfer of electrons. As a result, Ru^4+^-LiNi_0.5_Mn_1.5_O_4_ (Fd$\bar{3}$m, P4_3_32) all have a better electronic conductivity than LiNi_0.5_Mn_1.5_O_4_ (Fd$\bar{3}$m, P4_3_32). Thirdly, compared with Ni (3d^8^, 2 vacancies), Ru (4d^4^, 6 vacancies) has more outer vacancies and has a wider conduction band overlapping with the O 2p orbitals, which both contribute to enhancing the movement of electrons and lithium ions. It is worth noting that the LiNi_0.5_Mn_1.5_O_4_ cathode material has a tendency to be disordered as a result of Ru^4+^ doping. Disordered LiNi_0.5_Mn_1.5_O_4_ remains in a disordered state, and ordered LiNi_0.5_Mn_1.5_O_4_ transforms to semi-ordered LiNi_0.5_Mn_1.5_O_4_. Therefore, Ru^4+^-LiNi_0.5_Mn_1.5_O_4_ (P4_3_32) without an impurity phase has better high-rate properties than Ru^4+^-LiNi_0.5_Mn_1.5_O_4_ (Fd$\bar{3}$m).

## 4. Conclusions

With the introduction of Ru^4+^, the samples all deliver a higher discharge capacity, greater cycling stability, and better rate performance (especially at high charge–discharge current). Compared with LiNi_0.5_Mn_1.5_O_4_ (Fd$\bar{3}$m), the P4_3_32-structured cathode material shows the most obvious improvement in electrochemical performance after doping. After 300 cycles at a 1 C rate, the capacity retention of Ru^4+^-LiNi_0.5_Mn_1.5_O_4_ (P4_3_32) is still at 97.7% (at 1st cycle ≈132.8 mAh g^−1^; at 300th cycle ≈129.8 mAh g^−1^), which is larger than for undoped samples. Intriguingly, the specific capacity of Ru^4+^-LiNi_0.5_Mn_1.5_O_4_ (P4_3_32) remains at 100 mAh g^−1^ even at the extreme charge–discharge rate of 10 C (1470 mAh g^−1^). The introduction of Ru^4+^ hinders the ordering process of nickel/manganese ions during annealing treatment to suppress the lattice damage in the charge–discharge process. The stronger Ru-O bonding in the Ru^4+^-doped samples stabilizes the cathode structure and further improves the cycling stability. The greater number of O-Ru/Ni-O-Ru/Ni electron movement paths in the Ru^4+^-doped LiNi_0.5_Mn_1.5_O_4_ contribute to increasing the electron movement and enhancing the high-rate performance. After doping with Ru, disordered LiNi_0.5_Mn_1.5_O_4_ remains in a disordered state, and ordered LiNi_0.5_Mn_1.5_O_4_ is transformed into semi-ordered LiNi_0.5_Mn_1.5_O_4_ with no impurities, which explains why the electrochemical performance of Ru^4+^-LiNi_0.5_Mn_1.5_O_4_ (P4_3_32) is better than that of Ru^4+^-LiNi_0.5_Mn_1.5_O_4_ (Fd$\bar{3}$m). In terms of the low-cost solid-state synthesis method, Ru^4+^ doping of ordered LiNi_0.5_Mn_1.5_O_4_ provides a potential method of improving the electrochemical characteristics of high-voltage cathode materials for lithium-ion batteries.

## Data Availability

Not applicable.
